# Supplementary material for: Unbiased whole-genome deep sequencing of human and porcine stool samples reveals circulation of multiple groups of rotaviruses and a putative zoonotic infection
Source: Virus Evol. 2016 Oct 3;2(2):vew027. doi: 10.1093/ve/vew027 (PMC5522372; doi:10.1093/ve/vew027)
Supplement: Supplementary Data [file vew027_Supp.zip › Supplementary_Table_1.pdf]

Suppl Table 1

| Sample_ID | Sample collection date yyyy-mm-dd | Host | Host additional[a] | Province | Age of host | Age of host (months) | Sample material |
|---|---|---|---|---|---|---|---|
| 14152_1 | 2012-01-11 | Pig | Sow | Dong Thap | 30 m | 30 | Feces |
| 12070_96 | 2012-02-13 | Pig | Growing-Finising | Dong Thap | na[b] | | Feces |
| 14152_2 | 2012-02-13 | Pig | Growing-Finising | Dong Thap | na | | Feces |
| 14152_3 | 2012-02-13 | Pig | Weaner | Dong Thap | 1.5 m | 1.5 | Feces |
| 14152_4 | 2012-02-14 | Pig | Growing-Finising | Dong Thap | 3 m | 3 | Feces |
| 14152_5 | 2012-02-14 | Pig | Growing-Finising | Dong Thap | 2 m | 2 | Feces |
| 12070_1 | 2012-02-15 | Pig | Growing-Finising | Dong Thap | 3 m | 3 | Feces |
| 12070_2 | 2012-02-15 | Pig | Growing-Finising | Dong Thap | 3.5 m | 3.5 | Feces |
| 12070_3 | 2012-02-15 | Pig | Growing-Finising | Dong Thap | 4 m | 4 | Feces |
| 12070_4 | 2012-02-15 | Pig | Weaner | Dong Thap | 1 m | 1 | Feces |
| 12089_5 | 2012-02-15 | Pig | Sow + Pre-weaner | Dong Thap | 14 m | 14 | Feces |
| 12089_6 | 2012-02-15 | Pig | Sow + Pre-weaner | Dong Thap | 14 m | 14 | Feces |
| 14152_6 | 2012-02-15 | Pig | Sow | Dong Thap | 18 m | 18 | Boot swab |
| 14152_7 | 2012-02-15 | Pig | Growing-Finising | Dong Thap | 4 m | 4 | Feces |
| 14176_8 | 2012-02-15 | Pig | Weaner | Dong Thap | 1 m | 1 | Feces |
| 12089_7 | 2012-02-16 | Pig | Growing-Finising | Dong Thap | 2 m | 2 | Feces |
| 12089_8 | 2012-02-16 | Pig | Growing-Finising | Dong Thap | 2 m | 2 | Feces |
| 12089_9 | 2012-02-16 | Pig | Sow + Pre-weaner | Dong Thap | 24 m | 24 | Feces |
| 12089_10 | 2012-02-20 | Pig | Growing-Finising | Dong Thap | 2.5 m | 2.5 | Feces |
| 12089_12 | 2012-02-20 | Pig | Weaner | Dong Thap | 1 m | 1 | Feces |
| 14176_10 | 2012-02-20 | Pig | Growing-Finising | Dong Thap | 5 m | 5 | Feces |
| 14176_11 | 2012-02-20 | Pig | Weaner | Dong Thap | 1.5 m | 1.5 | Feces |
| 14176_12 | 2012-02-20 | Pig | Growing-Finising | Dong Thap | 6 m | 6 | Feces |
| 14176_13 | 2012-02-20 | Pig | Growing-Finising | Dong Thap | 4 m | 4 | Feces |
| 14176_9 | 2012-02-20 | Pig | Weaner | Dong Thap | 1 m | 1 | Feces |
| 12094 _11 | 2012-02-21 | Pig | Weaner | Dong Thap | 1.5 m | 1.5 | Feces |
| 12094 _13 | 2012-02-21 | Pig | Growing-Finising | Dong Thap | 5 m | 5 | Feces |
| 12094 _14 | 2012-02-21 | Pig | Sow + Pre-weaner | Dong Thap | na | | Feces |
| 14176_14 | 2012-02-21 | Pig | Gilt | Dong Thap | 5 m | 5 | Feces |
| 14177_15 | 2012-02-21 | Pig | Growing-Finising | Dong Thap | na | | Feces |
| 14177_16 | 2012-02-21 | Pig | Growing-Finising | Dong Thap | na | | Feces |
| 14177_17 | 2012-02-21 | Pig | Sow + Pre-weaner | Dong Thap | na | | Feces |
| 14177_18 | 2012-02-21 | Pig | Sow + Pre-weaner | Dong Thap | na | | Feces |
| 14177_19 | 2012-02-21 | Pig | Sow + Pre-weaner | Dong Thap | na | | Feces |
| 12094 _15 | 2012-02-22 | Pig | Sow + Pre-weaner | Dong Thap | 18 m | 18 | Feces |
| 12094 _16 | 2012-02-22 | Pig | Sow + Pre-weaner | Dong Thap | 24 m | 24 | Feces |
| 14177_20 | 2012-02-22 | Pig | Growing-Finising | Dong Thap | 2 m | 2 | Feces |
| 14177_21 | 2012-02-22 | Pig | Growing-Finising | Dong Thap | 2 m | 2 | Feces |
| 12094 _17 | 2012-03-05 | Pig | Gilt | Dong Thap | na | | Feces |
| 14174_29 | 2012-03-05 | Pig | Sow | Dong Thap | na | | Feces |
| 14174_30 | 2012-03-05 | Pig | Sow | Dong Thap | 36 m | 36 | Feces |
| 14174_31 | 2012-03-05 | Pig | Sow | Dong Thap | 36 m | 36 | Feces |
| 14174_32 | 2012-03-05 | Pig | Sow | Dong Thap | 36 m | 36 | Feces |
| 14174_33 | 2012-03-05 | Pig | Growing-Finising | Dong Thap | 4 m | 4 | Feces |
| 14174_34 | 2012-03-05 | Pig | Growing-Finising | Dong Thap | 4 m | 4 | Feces |
| 14174_35 | 2012-03-05 | Pig | Growing-Finising | Dong Thap | 4 m | 4 | Feces |
| 14175_22 | 2012-03-05 | Pig | Weaner | Dong Thap | na | | Feces |
| 14175_23 | 2012-03-05 | Pig | Growing-Finising | Dong Thap | na | | Feces |
| 14175_24 | 2012-03-05 | Pig | Sow + Pre-weaner | Dong Thap | na | | Feces |
| 14175_25 | 2012-03-05 | Pig | Gilt | Dong Thap | na | | Feces |

| | | | | | | | |
|---|---|---|---|---|---|---|---|
| 14175_26 | 2012-03-05 | Pig | Growing-Finising | Dong Thap | 7 m | 7 | Feces |
| 14175_27 | 2012-03-05 | Pig | Growing-Finising | Dong Thap | 7 m | 7 | Feces |
| 14175_28 | 2012-03-05 | Pig | Growing-Finising | Dong Thap | 7 m | 7 | Feces |
| 12088_31 | 2012-03-06 | Pig | Growing-Finising | Dong Thap | 3.5 m | 3.5 | Feces |
| 12088_32 | 2012-03-06 | Pig | Sow + Pre-weaner | Dong Thap | 38 m | 38 | Feces |
| 12088_33 | 2012-03-06 | Pig | Sow + Pre-weaner | Dong Thap | 38 m | 38 | Feces |
| 12094 _18 | 2012-03-06 | Pig | Growing-Finising | Dong Thap | na | | Feces |
| 14226_42 | 2012-03-06 | Pig | Sow + Pre-weaner | Dong Thap | 38 m | 38 | Feces |
| 14225_43 | 2012-03-06 | Pig | Sow + Pre-weaner | Dong Thap | 38 m | 38 | Feces |
| 14225_44 | 2012-03-06 | Pig | Sow + Pre-weaner | Dong Thap | 38 m | 38 | Feces |
| 14226_36 | 2012-03-06 | Pig | Growing-Finising | Dong Thap | 2 m | 2 | Feces |
| 14226_37 | 2012-03-06 | Pig | Growing-Finising | Dong Thap | 2 m | 2 | Feces |
| 14226_38 | 2012-03-06 | Pig | Sow + Pre-weaner | Dong Thap | 15 m | 15 | Feces |
| 14226_39 | 2012-03-06 | Pig | Sow + Pre-weaner | Dong Thap | 15 m | 15 | Feces |
| 14226_40 | 2012-03-06 | Pig | Growing-Finising | Dong Thap | 3.5 m | 3.5 | Feces |
| 14226_41 | 2012-03-06 | Pig | Growing-Finising | Dong Thap | 3.5 m | 3.5 | Feces |
| 12088_34 | 2012-03-07 | Pig | Growing-Finising | Dong Thap | na | | Feces |
| 12088_35 | 2012-03-07 | Pig | Growing-Finising | Dong Thap | na | | Feces |
| 14225_45 | 2012-03-07 | Pig | Weaner | Dong Thap | na | | Feces |
| 14225_46 | 2012-03-07 | Pig | Weaner | Dong Thap | na | | Feces |
| 14225_47 | 2012-03-07 | Pig | Growing-Finising | Dong Thap | na | | Feces |
| 12088_36 | 2012-03-08 | Pig | Growing-Finising | Dong Thap | 6 m | 6 | Feces |
| 12088_37 | 2012-03-08 | Pig | Growing-Finising | Dong Thap | 5 m | 5 | Feces |
| 14150_50 | 2012-03-08 | Pig | Growing-Finising | Dong Thap | na | | Feces |
| 14225_48 | 2012-03-08 | Pig | Growing-Finising | Dong Thap | 6 m | 6 | Feces |
| 14225_49 | 2012-03-08 | Pig | Pre-weaner | Dong Thap | na | | Feces |
| 12087_38 | 2012-03-12 | Pig | Sow | Dong Thap | 11 m | 11 | Feces |
| 12087_39 | 2012-03-12 | Pig | Growing-Finising | Dong Thap | 6 m | 6 | Feces |
| 12087_40 | 2012-03-12 | Pig | Weaner | Dong Thap | 1 m | 1 | Feces |
| 14150_51 | 2012-03-12 | Pig | Weaner | Dong Thap | 1.5 m | 1.5 | Feces |
| 14150_52 | 2012-03-12 | Pig | Sow | Dong Thap | 12 m | 12 | Feces |
| 14150_53 | 2012-03-12 | Pig | Weaner | Dong Thap | 1 m | 1 | Feces |
| 14150_54 | 2012-03-12 | Pig | Weaner | Dong Thap | 1 m | 1 | Feces |
| 14150_55 | 2012-03-12 | Pig | Weaner | Dong Thap | 1 m | 1 | Feces |
| 12087_41 | 2012-03-14 | Pig | Growing-Finising | Dong Thap | 1.5 m | 1.5 | Feces |
| 12087_42 | 2012-03-14 | Pig | Weaner | Dong Thap | 1.5 m | 1.5 | Feces |
| 14150_56 | 2012-03-14 | Pig | Growing-Finising | Dong Thap | 5.5 m | 5.5 | Feces |
| 14151_57 | 2012-03-14 | Pig | Growing-Finising | Dong Thap | 5.5 m | 5.5 | Feces |
| 14151_58 | 2012-03-14 | Pig | Weaner | Dong Thap | 1 m | 1 | Feces |
| 14151_59 | 2012-03-14 | Pig | Weaner | Dong Thap | 1 m | 1 | Feces |
| 14151_60 | 2012-03-14 | Pig | Gilt | Dong Thap | 4.5 m | 4.5 | Feces |
| 14151_61 | 2012-03-14 | Pig | Growing-Finising | Dong Thap | 1.5 m | 1.5 | Feces |
| 14151_62 | 2012-03-14 | Pig | Weaner | Dong Thap | 1.5 m | 1.5 | Feces |
| 12087_43 | 2012-03-15 | Pig | Growing-Finising | Dong Thap | 3 m | 3 | Feces |
| 14151_63 | 2012-03-15 | Pig | Gilt | Dong Thap | 7 m | 7 | Feces |
| 14254_1 | 2012-03-15 | Pig | Growing-Finising | Dong Thap | 3 m | 3 | Feces |
| 14254_2 | 2012-03-15 | Pig | Growing-Finising | Dong Thap | na | | Feces |
| 14254_3 | 2012-03-15 | Pig | Growing-Finising | Dong Thap | na | | Feces |
| 14254_4 | 2012-03-15 | Pig | Sow + Pre-weaner | Dong Thap | na | | Feces |
| 12087_44 | 2012-03-19 | Pig | Gilt | Dong Thap | 2 m | 2 | Feces |
| 14250_8 | 2012-03-20 | Pig | Sow + Pre-weaner | Dong Thap | 24 m | 24 | Feces |
| 14250_9 | 2012-03-20 | Pig | Weaner | Dong Thap | 1 m | 1 | Feces |
| 14254_5 | 2012-03-20 | Pig | Weaner | Dong Thap | 2 m | 2 | Feces |

| | | | | | | | |
|---|---|---|---|---|---|---|---|
| 14254_6 | 2012-03-20 | Pig | Weaner | Dong Thap | 2 m | 2 | Feces |
| 14254_7 | 2012-03-20 | Pig | Sow + Pre-weaner | Dong Thap | 12 m | 12 | Feces |
| 12129_45 | 2012-03-21 | Pig | Growing-Finising | Dong Thap | 2 m | 2 | Feces |
| 12129_46 | 2012-03-21 | Pig | Growing-Finising | Dong Thap | 4 m | 4 | Feces |
| 14250_10 | 2012-03-21 | Pig | Growing-Finising | Dong Thap | 2 m | 2 | Feces |
| 14250_11 | 2012-03-21 | Pig | Growing-Finising | Dong Thap | 2 m | 2 | Feces |
| 14250_12 | 2012-03-21 | Pig | Sow | Dong Thap | 18 m | 18 | Boot swab |
| 14250_13 | 2012-03-21 | Pig | Weaner | Dong Thap | 1.5 m | 1.5 | Feces |
| 14250_14 | 2012-03-21 | Pig | Weaner | Dong Thap | 1.5 m | 1.5 | Feces |
| 14302_15 | 2012-03-21 | Pig | Growing-Finising | Dong Thap | 4 m | 4 | Feces |
| 14302_16 | 2012-03-26 | Pig | Weaner | Dong Thap | 1.5 m | 1.5 | Feces |
| 14302_17 | 2012-03-26 | Pig | Weaner | Dong Thap | 1.5 m | 1.5 | Feces |
| 14302_18 | 2012-03-26 | Pig | Growing-Finising | Dong Thap | 3 m | 3 | Feces |
| 12129_47 | 2012-03-27 | Pig | Sow + Pre-weaner | Dong Thap | 36 m | 36 | Feces |
| 12129_48 | 2012-03-27 | Pig | Sow + Pre-weaner | Dong Thap | 36 m | 36 | Feces |
| 12129_49 | 2012-03-27 | Pig | Sow + Pre-weaner | Dong Thap | 36 m | 36 | Feces |
| 12129_50 | 2012-03-27 | Pig | Sow + Pre-weaner | Dong Thap | 48 m | 48 | Feces |
| 12129_51 | 2012-03-27 | Pig | Weaner | Dong Thap | 1 m | 1 | Feces |
| 12130_52 | 2012-03-27 | Pig | Weaner | Dong Thap | 1 m | 1 | Feces |
| 14249_22 | 2012-03-27 | Pig | Growing-Finising | Dong Thap | 2.5 m | 2.5 | Feces |
| 14249_23 | 2012-03-27 | Pig | Sow + Pre-weaner | Dong Thap | 36 m | 36 | Feces |
| 14249_24 | 2012-03-27 | Pig | Growing-Finising | Dong Thap | 4.5 m | 4.5 | Feces |
| 14302_19 | 2012-03-27 | Pig | Growing-Finising | Dong Thap | 3 m | 3 | Feces |
| 14302_20 | 2012-03-27 | Pig | Growing-Finising | Dong Thap | 3 m | 3 | Feces |
| 14302_21 | 2012-03-27 | Pig | Growing-Finising | Dong Thap | 4 m | 4 | Feces |
| 12130_53 | 2012-04-03 | Pig | Weaner | Dong Thap | 1.5 m | 1.5 | Feces |
| 14249_25 | 2012-04-03 | Pig | Weaner | Dong Thap | 1.5 m | 1.5 | Feces |
| 14249_26 | 2012-04-03 | Pig | Weaner | Dong Thap | 1.5 m | 1.5 | Feces |
| 12130_54 | 2012-04-04 | Pig | Growing-Finising | Dong Thap | 2.5 m | 2.5 | Feces |
| 12130_55 | 2012-04-04 | Pig | Growing-Finising | Dong Thap | 3 m | 3 | Feces |
| 12130_56 | 2012-04-04 | Pig | Growing-Finising | Dong Thap | 3 m | 3 | Feces |
| 14249_27 | 2012-04-04 | Pig | Growing-Finising | Dong Thap | 1.5 m | 1.5 | Feces |
| 14249_28 | 2012-04-04 | Pig | Growing-Finising | Dong Thap | 2.5 m | 2.5 | Feces |
| 14249_29 | 2012-04-04 | Pig | Growing-Finising | Dong Thap | 3 m | 3 | Feces |
| 12144_57 | 2012-04-05 | Pig | Growing-Finising | Dong Thap | 3 m | 3 | Feces |
| 14280_30 | 2012-04-05 | Pig | Weaner | Dong Thap | 1.1 m | 1.1 | Feces |
| 14280_31 | 2012-04-05 | Pig | Sow + Pre-weaner | Dong Thap | 24 m | 24 | Feces |
| 14280_32 | 2012-04-05 | Pig | Sow + Pre-weaner | Dong Thap | 24 m | 24 | Feces |
| 14280_33 | 2012-04-05 | Pig | Growing-Finising | Dong Thap | 5 m | 5 | Feces |
| 14280_34 | 2012-04-05 | Pig | Growing-Finising | Dong Thap | 6 m | 6 | Feces |
| 12144_58 | 2012-04-09 | Pig | Weaner | Dong Thap | 0.1 m | 0.1 | Feces |
| 12144_59 | 2012-04-09 | Pig | Growing-Finising | Dong Thap | 5 m | 5 | Feces |
| 14280_35 | 2012-04-09 | Pig | Weaner | Dong Thap | 0.1 m | 0.1 | Feces |
| 14280_36 | 2012-04-09 | Pig | Weaner | Dong Thap | 1 m | 1 | Feces |
| 12144_60 | 2012-04-10 | Pig | Growing-Finising | Dong Thap | 4 m | 4 | Feces |
| 12144_61 | 2012-04-10 | Pig | Sow | Dong Thap | 20 m | 20 | Feces |
| 14280_37 | 2012-04-10 | Pig | Growing-Finising | Dong Thap | 8 m | 8 | Feces |
| 12013_46 | 2012-10-26 | Human | | Dong Thap | 5 | 60 | Feces |
| 12013_45 | 2012-10-31 | Human | | Dong Thap | 13 | 156 | Feces |
| 12013_47 | 2012-11-01 | Human | | Dong Thap | 89 | 1068 | Feces |
| 12013_48 | 2012-11-01 | Human | | Dong Thap | 11 | 132 | Feces |
| 12013_49 | 2012-11-21 | Human | | Dong Thap | 21 | 252 | Feces |
| 12013_50 | 2012-11-21 | Human | | Dong Thap | 5 | 60 | Feces |

| | | | | | | |
|---|---|---|---|---|---|---|
| 12013_51 | 2012-11-21 | Human | Dong Thap | 9 | 108 | Feces |
| 12053_52 | 2012-11-26 | Human | Dong Thap | 4 | 48 | Feces |
| 12053_53 | 2012-11-26 | Human | Dong Thap | 5 | 60 | Feces |
| 12053_54 | 2012-11-27 | Human | Dong Thap | 11 | 132 | Feces |
| 12053_55 | 2012-11-28 | Human | Dong Thap | 11 | 132 | Feces |
| 12053_56 | 2012-11-28 | Human | Dong Thap | 13 | 156 | Feces |
| 12053_57 | 2012-12-05 | Human | Dong Thap | 10 | 120 | Feces |
| 12053_58 | 2012-12-05 | Human | Dong Thap | 6 | 72 | Feces |
| 12035_59 | 2012-12-07 | Human | Vinh Long | 13 | 156 | Feces |
| 12035_61 | 2012-12-10 | Human | Dong Thap | 9 | 108 | Feces |
| 12035_60 | 2012-12-12 | Human | Dong Thap | 11 | 132 | Feces |
| 12035_62 | 2012-12-12 | Human | Dong Thap | 13 | 156 | Feces |
| 12035_63 | 2012-12-14 | Human | Dong Thap | 14 | 168 | Feces |
| 12035_64 | 2012-12-17 | Human | Dong Thap | 11 | 132 | Feces |
| 12034_66 | 2012-12-20 | Human | Dong Thap | 11 | 132 | Feces |
| 12035_65 | 2012-12-20 | Human | Dong Thap | 9 | 108 | Feces |
| 12034_67 | 2012-12-24 | Human | Dong Thap | 11 | 132 | Feces |
| 12034_68 | 2012-12-24 | Human | Dong Thap | 10 | 120 | Feces |
| 12034_69 | 2012-12-25 | Human | Dong Thap | 14 | 168 | Feces |
| 12034_70 | 2012-12-28 | Human | Dong Thap | 18 | 216 | Feces |
| 12034_71 | 2013-01-03 | Human | Dong Thap | 5 | 60 | Feces |
| 12034_72 | 2013-01-03 | Human | Dong Thap | 9 | 108 | Feces |
| 12056_73 | 2013-01-11 | Human | Dong Thap | 77 | 924 | Feces |
| 12056_74 | 2013-01-16 | Human | Dong Thap | 10 | 120 | Feces |
| 12056_75 | 2013-01-18 | Human | Dong Thap | 9 | 108 | Feces |
| 12056_76 | 2013-01-21 | Human | Dong Thap | 9 | 108 | Feces |
| 12056_77 | 2013-01-24 | Human | Dong Thap | 7 | 84 | Feces |
| 12056_78 | 2013-01-29 | Human | Dong Thap | 8 | 96 | Feces |
| 12056_79 | 2013-01-31 | Human | Dong Thap | 18 | 216 | Feces |
| 12057_80 | 2013-02-01 | Human | Dong Thap | 7 | 84 | Feces |
| 12057_81 | 2013-02-01 | Human | Dong Thap | 11 | 132 | Feces |
| 12057_82 | 2013-02-04 | Human | Dong Thap | 5 | 60 | Feces |
| 12057_83 | 2013-02-19 | Human | Dong Thap | 4 | 48 | Feces |
| 12057_85 | 2013-02-22 | Human | Dong Thap | 7 | 84 | Feces |
| 12057_86 | 2013-02-26 | Human | Dong Thap | 16 | 192 | Feces |
| 12067_87 | 2013-02-26 | Human | Dong Thap | ***6*** | 72 | Feces |
| 12067_88 | 2013-02-26 | Human | Dong Thap | 7 | 84 | Feces |
| 12057_84 | 2013-02-27 | Human | Dong Thap | 81 | 972 | Feces |
| 12067_89 | 2013-03-20 | Human | Dong Thap | 14 | 168 | Feces |
| 12067_90 | 2013-03-21 | Human | Dong Thap | 19 | 228 | Feces |
| 12067_91 | 2013-04-01 | Human | Dong Thap | 10 | 120 | Feces |
| 12067_92 | 2013-04-03 | Human | Dong Thap | 7 | 84 | Feces |
| 12067_93 | 2013-04-05 | Human | Dong Thap | 11 | 132 | Feces |
| 12070_94 | 2013-04-10 | Human | Dong Thap | 5 | 60 | Feces |
| 16020_1 | 2013-06-17 | Human | Dong Thap | 5 m | 5 | Feces |
| 16020_2 | 2013-06-18 | Human | Dong Thap | 72 | 864 | Feces |
| 16020_3 | 2013-06-18 | Human | Dong Thap | 8 m | 8 | Feces |
| 16020_4 | 2013-06-18 | Human | Dong Thap | 44 | 528 | Feces |
| 16020_5 | 2013-06-25 | Human | Dong Thap | 6 m | 6 | Feces |
| 16020_6 | 2013-06-26 | Human | Dong Thap | 2 m | 2 | Feces |
| 16020_7 | 2013-06-26 | Human | Dong Thap | **54** | 648 | Feces |
| 16020_10 | 2013-06-27 | Human | Dong Thap | 5 | 60 | Feces |
| 16020_8 | 2013-06-27 | Human | Dong Thap | 35 | 420 | Feces |

| | | | | | | |
|---|---|---|---|---|---|---|
| 16020_9 | 2013-06-27 | Human | Dong Thap | 1 | 12 | Feces |
| 16020_11 | 2013-07-02 | Human | Dong Thap | 9 m | 9 | Feces |
| 16020_12 | 2013-07-02 | Human | Dong Thap | 9 m | 9 | Feces |
| 16020_13 | 2013-07-04 | Human | Dong Thap | 40 | 480 | Feces |
| 16020_14 | 2013-07-04 | Human | Dong Thap | 64 | 768 | Feces |
| 16020_15 | 2013-07-08 | Human | Dong Thap | 1 | 12 | Feces |
| 16020_16 | 2013-07-08 | Human | Dong Thap | 7 m | 7 | Feces |
| 16020_17 | 2013-07-08 | Human | Dong Thap | 7 | 84 | Feces |
| 16020_18 | 2013-07-09 | Human | Dong Thap | 88 | 1056 | Feces |
| 16020_19 | 2013-07-18 | Human | Dong Thap | 8 m | 8 | Feces |
| 16020_20 | 2013-07-19 | Human | Dong Thap | 68 | 816 | Feces |
| 16020_22 | 2013-07-20 | Human | Dong Thap | 75 | 900 | Feces |
| 16020_21 | 2013-07-22 | Human | Dong Thap | 83 | 996 | Feces |
| 16020_23 | 2013-07-22 | Human | Dong Thap | 80 | 960 | Feces |
| 16020_24 | 2013-07-25 | Human | Dong Thap | 81 | 972 | Feces |
| 16020_25 | 2013-07-25 | Human | Dong Thap | 1 | 12 | Feces |
| 16020_26 | 2013-07-30 | Human | Dong Thap | 33 | 396 | Feces |
| 16020_27 | 2013-07-31 | Human | Dong Thap | 5 | 60 | Feces |
| 16020_28 | 2013-08-02 | Human | Dong Thap | 71 | 852 | Feces |
| 16020_29 | 2013-08-07 | Human | Dong Thap | 1 | 12 | Feces |
| 16020_30 | 2013-08-08 | Human | Dong Thap | 8 | 96 | Feces |
| 16020_31 | 2013-08-21 | Human | Dong Thap | 48 | 576 | Feces |
| 16020_32 | 2013-08-22 | Human | Dong Thap | 7 m | 7 | Feces |
| 16020_33 | 2013-08-22 | Human | Dong Thap | 82 | 984 | Feces |
| 16020_34 | 2013-08-23 | Human | Dong Thap | 1 | 12 | Feces |
| 16020_35 | 2013-08-27 | Human | Dong Thap | 1 | 12 | Feces |
| 16020_36 | 2013-08-28 | Human | Dong Thap | 10 m | 10 | Feces |
| 16020_37 | 2013-08-28 | Human | Dong Thap | 9 m | 9 | Feces |
| 16020_38 | 2013-09-09 | Human | Dong Thap | 1 | 12 | Feces |
| 16020_39 | 2013-09-10 | Human | Dong Thap | **57** | 684 | Feces |
| 16020_40 | 2013-09-16 | Human | Dong Thap | 17 | 204 | Feces |
| 16020_41 | 2013-09-18 | Human | Dong Thap | 45 | 540 | Feces |
| 16020_42 | 2013-09-22 | Human | Dong Thap | 93 | 1116 | Feces |
| 16020_43 | 2013-09-23 | Human | Dong Thap | 1 | 12 | Feces |
| 16020_44 | 2013-09-23 | Human | Dong Thap | **53** | 636 | Feces |
| 16020_45 | 2013-09-24 | Human | Dong Thap | 1 | 12 | Feces |
| 16020_46 | 2013-09-25 | Human | Dong Thap | 47 | 564 | Feces |
| 16020_47 | 2013-09-25 | Human | Dong Thap | 55 | 660 | Feces |
| 16020_48 | 2013-10-02 | Human | Dong Thap | 1 | 12 | Feces |
| 16020_49 | 2013-10-03 | Human | Dong Thap | 11 m | 11 | Feces |
| 16020_50 | 2013-10-03 | Human | Dong Thap | 9 m | 9 | Feces |
| 16020_52 | 2013-10-11 | Human | Dong Thap | 83 | 996 | Feces |
| 16020_53 | 2013-10-11 | Human | Dong Thap | 7 m | 7 | Feces |
| 16020_51 | 2013-10-14 | Human | Dong Thap | 24 | 288 | Feces |
| 16020_54 | 2013-10-14 | Human | Dong Thap | 1 | 12 | Feces |
| 16020_55 | 2013-10-14 | Human | Dong Thap | 64 | 768 | Feces |
| 16020_56 | 2013-10-18 | Human | Dong Thap | 10 | 120 | Feces |
| 16020_57 | 2013-10-22 | Human | Dong Thap | 57 | 684 | Feces |
| 16020_58 | 2013-10-24 | Human | Dong Thap | 1 | 12 | Feces |
| 16020_59 | 2013-10-24 | Human | Dong Thap | 1 | 12 | Feces |
| 16020_60 | 2013-10-29 | Human | Dong Thap | 84 | 1008 | Feces |
| 16020_61 | 2013-10-29 | Human | Dong Thap | 1 | 12 | Feces |
| 16020_62 | 2013-10-31 | Human | Dong Thap | 46 | 552 | Feces |

| | | | | | | |
|---|---|---|---|---|---|---|
| 16020_63 | 2013-11-01 | Human | Dong Thap | 23 | 276 | Feces |
| 16020_64 | 2013-11-04 | Human | Dong Thap | 1 | 12 | Feces |
| 16020_65 | 2013-11-05 | Human | Dong Thap | 65 | 780 | Feces |
| 16020_66 | 2013-11-06 | Human | Dong Thap | 61 | 732 | Feces |
| 16020_67 | 2013-11-11 | Human | Dong Thap | 22 | 264 | Feces |
| 16020_68 | 2013-11-18 | Human | Dong Thap | 56 | 672 | Feces |
| 16020_69 | 2013-11-21 | Human | Dong Thap | 5 m | 5 | Feces |
| 16020_70 | 2013-11-22 | Human | Dong Thap | 10 m | 10 | Feces |
| 16020_71 | 2013-11-22 | Human | Dong Thap | 1 | 12 | Feces |
| 16020_72 | 2013-11-22 | Human | Dong Thap | 1 | 12 | Feces |
| 16020_73 | 2013-11-28 | Human | Dong Thap | 10 m | 10 | Feces |
| 16020_74 | 2013-11-29 | Human | Dong Thap | 1 | 12 | Feces |
| 16020_75 | 2013-11-29 | Human | Dong Thap | 10 m | 10 | Feces |
| 16020_76 | 2013-12-12 | Human | Dong Thap | 63 | 756 | Feces |
| 16020_77 | 2013-12-12 | Human | Dong Thap | 2 | 24 | Feces |
| 16020_78 | 2013-12-12 | Human | Dong Thap | 1 | 12 | Feces |
| 16020_79 | 2013-12-13 | Human | Dong Thap | 2 | 24 | Feces |
| 16020_80 | 2013-12-19 | Human | Dong Thap | 7 m | 7 | Feces |
| 16020_81 | 2013-12-20 | Human | Dong Thap | 11 m | 11 | Feces |
| 16020_82 | 2013-12-24 | Human | Dong Thap | 1 | 12 | Feces |
| 16020_83 | 2013-12-24 | Human | Dong Thap | 1 | 12 | Feces |
| 16020_84 | 2013-12-25 | Human | Dong Thap | 3 | 36 | Feces |
| 16020_85 | 2013-12-26 | Human | Dong Thap | 1 | 12 | Feces |
| 16020_86 | 2013-12-30 | Human | Dong Thap | 68 | 816 | Feces |
| 16020_87 | 2014-01-01 | Human | Dong Thap | 6 m | 6 | Feces |
| 16020_88 | 2014-01-01 | Human | Dong Thap | 7 m | 7 | Feces |
| 16020_89 | 2014-01-02 | Human | Dong Thap | 52 | 624 | Feces |
| 16020_90 | 2014-01-09 | Human | Dong Thap | 1 | 12 | Feces |
| 16020_91 | 2014-01-13 | Human | Dong Thap | 1 | 12 | Feces |
| 16020_92 | 2014-01-14 | Human | Dong Thap | 9 m | 9 | Feces |
| 16020_93 | 2014-01-16 | Human | Dong Thap | 1 | 12 | Feces |
| 16020_94 | 2014-01-16 | Human | Dong Thap | 2 | 24 | Feces |
| 16020_95 | 2014-01-20 | Human | Dong Thap | 1 | 12 | Feces |
| 16020_96 | 2014-01-21 | Human | Dong Thap | 1 | 12 | Feces |

Footnote a: Additional details of pig hosts
Footnote b: na = not available
