# Supplementary material for: Unbiased whole-genome deep sequencing of human and porcine stool samples reveals circulation of multiple groups of rotaviruses and a putative zoonotic infection
Source: Virus Evol. 2016 Oct 3;2(2):vew027. doi: 10.1093/ve/vew027 (PMC5522372; doi:10.1093/ve/vew027)
Supplement: Supplementary Data [file vew027_Supp.zip › SuppS2.pdf]

Suppl Figure 2

RVA genotype constellation

Strain ID	VP7	VP4	VP6	VP1	VP2	VP3	NSP1	NSP2	NSP3	NSP4	NSP5
12070_4	G1/G4	P[8]	I1	R1	C1	M1	A1/A8	N1/N1	T1/T1	E1/E1	H1/H1
12129_48	G4	P[6]	I1	R1	C1	M1	A8	N1	T1	E1	H1
12129_49	G4	P[6]	I1	R1	C1	M1	A8	N1	T1	E1	H1
14150_53	G9/G11	P[13]/P[23]	I5/I5	R1/R1	C1	M1	A8/A8	N1/N1	T1/T7	E1/E1	H1
14150_54	G11	P[13]	I5	R1	C1	M1	A8	N1	T7	E1	H1
14175_24	G9	P[23]	I5	R1	C1	M1	A8	N1	T1	E1	H1
14225_44	G4	P[6]	I1	R1	C1	M1	A8	N1	T1	E1	H1
14225_45	G5	P[13]	I5	R1	C1	M1	A8	N1	T7	E1	H1
14225_46	G5	P[13]	I5	R1	C1	M1	A8	N1	T7		H1
14226_39	G4	P[6]	I1	R1	C1	M1	A8	N1	T1	E1	H1
14226_42	G4	P[6]	I1	R1	C1	M1	A8	N1	T1	E1	H1
14249_23	G4	P[6]	I1	R1	C1	M1	A8	N1	T1	E1	H1
14250_9	G4	P[6]	I1	R1	C1	M1	A8	N1	T7	E1	H1
12013_46	G1/G2	P[8]/P[4]	I1/I2	R1	C1/C2	M1/M2	A1/A2	N1/N2	T1/T2	E1/E2	H1/H2
12013_45	G2	P[4]	I2	R2	C2	M2	A2	N2	T2	E2	H2
12013_49	G1	P[8]	I1	R1	C1	M1	A1	N1	T1	E1	H1
12013_50	G1	P[8]	I1	R1	C1	M1	A1	N1	T1	E1	H1
12013_51	G1	P[8]	I1	R1	C1	M1	A1	N1	T1	E1	H1
12053_52	G1	P[8]	I1	R1	C1	M1	A1	N1	T1	E1	H1
12053_53	G1	P[8]	I1	R1	C1	M1	A1	N1	T1	E1	H1
12053_54	G1	P[8]	I1	R1	C1	M1	A1	N1	T1	E1	H1
12053_55	G1	P[8]	I1	R1	C1	M1	A1	N1	T1	E1	H1
12053_56	G1	P[8]	I1	R1	C1	M1	A1	N1	T1	E1	H1
12053_57	G1	P[8]	I1	R1	C1	M1	A1	N1	T1	E1	H1
12053_58	G1	P[8]	I1	R1	C1	M1	A1	N1	T1	E1	H1
12035_61	G1	P[8]	I1	R1	C1	M1	A1	N1	T1	E1	H1
12035_62	G1	P[8]	I1	R1	C1	M1	A1	N1	T1	E1	H1
12035_63	G1	P[8]	I1	R1	C1	M1	A1	N1	T1	E1	H1
12035_64	G1	P[8]	I1	R1	C1	M1	A1	N1	T1	E1	H1
12035_65	G1	P[8]	I1	R1	C1	M1	A1	N1	T1	E1	H1
12034_66	G1	P[8]	I1	R1	C1	M1	A1	N1	T1	E1	H1
12034_67	G1	P[8]	I1	R1	C1	M1	A1	N1	T1	E1	H1
12034_68	G1/G2	P[8]/P[4]	I1/I2	R1/R2	C1	M1/M2	A1/A2	N1/N2	T1/T2	E1/E2	H1
12034_69	G1	P[8]	I1	R1	C1	M1	A1	N1/N1	T1	E1	H1
12034_70	G1	P[8]	I1	R1	C1	M1	A1	N1	T1	E1	H1
12034_72	G1	P[8]	I1	R1	C1	M1	A1	N1	T1	E1	H1

RVA genotype constellation

Strain ID	VP7	VP4	VP6	VP1	VP2	VP3	NSP1	NSP2	NSP3	NSP4	NSP5
12056_74	G1	P[8]	I1	R1	C1	M1	A1	N1	T1	E1	H1
12056_79	G1/G2	P[8]/P[4]	I1/I2	R1/R2	C1/C2	M1/M2	A1/A2	N1/N2	T1/T2	E2	H2
12057_80	G1	P[8]	I1	R1	C1	M1	A1	N1	T1	E1	H1
12057_81	G1	P[8]	I1	R1	C1	M1	A1	N1	T1	E1	H1
12057_82	G1	P[8]	I1	R1	C1	M1	A1	N1	T1	E1	H1
12057_83	G1	P[8]	I1	R1	C1	M1	A1	N1	T1	E1	H1
12057_85	G1	P[8]	I1	R1	C1	M1	A1	N1	T1	E1	H1
12057_86	G1/G2	P[8]/P[4]	I1/I2	R1/R2	C1/C2	M1/M2	A1/A2	N1/N2	T1/T2	E1/E2	H1
12067_87	G1/G2	P[8]/P[4]	I1/I2	R1/R2	C1/C2	M1/M2	A1/A2	N1/N2	T1/T2	E1/E2	H1/H2
12067_88	G1	P[8]	I1	R1	C1	M1	A1	N1	T1	E1	H1
12067_91	G1/G2	P[8]/P[4]	I1/I2	R2	C1/C2	M2	A1/A2	N1/N2	T1/T2	E1/E2	H1/H2
12067_93	G1	P[8]	I1	R1	C1	M1	A1	N1	T1	E1	H1
12070_94	G1	P[8]	I1	R1	C1	M1	A1	N1	T1	E1	H1
16020_5	G1	P[8]	I1	R1	C1	M1	A1	N1	T1	E1	H1
16020_7	G4	P[6]	I1	R1	C1	M1	A8	N1	T7	E1	H1
16020_9	G1	P[8]	I1	R1	C1	M1	A1	N1	T1	E1	H1
16020_11	G2	P[8]	I2	R2	C2	M2	A2	N2	T2	E2	H2
16020_19	G2	P[4]	I2	R2	C2	M2	A2	N2	T2	E2	H2
16020_32	G1	P[8]		R1	C1	M1	A1	N1	T1	E1	H1
16020_35	G1	P[8]	I1	R1	C1	M1	A1	N1	T1	E1	H1
16020_36	G1	P[8]	I1	R1	C1	M1	A1	N1	T1	E1	H1
16020_37	G1	P[8]	I1	R1	C1	M1	A1	N1	T1	E1	H1
16020_39	G2	P[4]	I2	R2	C2	M2	A2	N2	T2	E2	H2
16020_44	G2	P[4]	I2	R2	C2	M2	A2	N2	T2	E2	H2
16020_45	G2	P[4]	I2	R2	C2	M2	A2	N2	T2	E2	H2
16020_49	G2	P[4]	I2	R2	C2	M2	A2	N2	T2	E2	H2
16020_72	G1	P[8]	I2	R2	C2	M2	A2	N2	T2	E2	H2
16020_74	G2	P[4]	I2	R2	C2	M2	A2	N2	T2	E2	H2
16020_77	G2	P[4]	I2	R2	C2	M2	A2	N2	T2	E2	H2
16020_80	G1	P[8]	I2	R2	C2	M2	A2	N2	T2	E2	H2
16020_81	G2	P[4]	I2	R2	C2	M2	A2	N2	T2	E2	H2
16020_83	G3	P[8]	I1	R1	C1	M1	A1	N1	T1	E1	H1
16020_91	G2	P[4]	I2	R2	C2	M2	A2	N2	T2	E2	H2
16020_92	G1	P[8]	I2	R2	C2	M2	A2	N2	T2	E2	H2
16020_93	G2	P[4]	I2	R2	C2	M2	A2	N2	T2	E2	H2
16020_95	G3	P[8]	I1	R1	C1	M1	A1	N1	T1	E1	H1
16020_96	G2	P[4]	I2	R2	C2	M2	A2	N2	T2	E2	H2
