# Supplementary material for: Unbiased whole-genome deep sequencing of human and porcine stool samples reveals circulation of multiple groups of rotaviruses and a putative zoonotic infection
Source: Virus Evol. 2016 Oct 3;2(2):vew027. doi: 10.1093/ve/vew027 (PMC5522372; doi:10.1093/ve/vew027)

Suppl Figure 3A

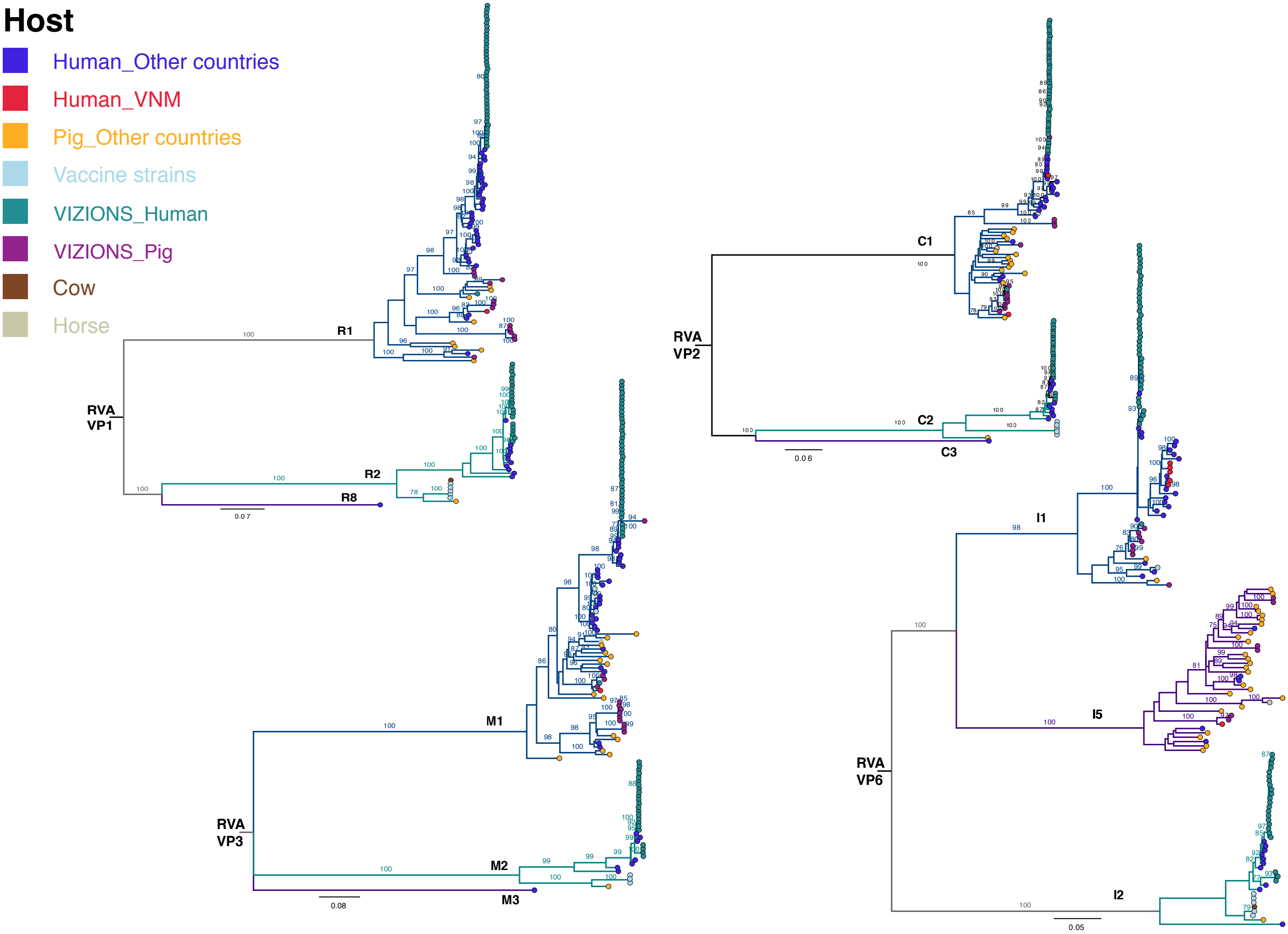

Supplement: Supplementary Data [file vew027_Supp.zip › SuppS3A.pdf]
