# Supplementary material for: Unbiased whole-genome deep sequencing of human and porcine stool samples reveals circulation of multiple groups of rotaviruses and a putative zoonotic infection
Source: Virus Evol. 2016 Oct 3;2(2):vew027. doi: 10.1093/ve/vew027 (PMC5522372; doi:10.1093/ve/vew027)
Supplement: Supplementary Data [file vew027_Supp.zip › VEVOLU-2016-022_supplementary_data_revised.docx]

**My VT Phan et al. Rotavirus genomes and diversity in human and pigs**

Supplementary Data

**Supplementary Table 1.** The collection dates and ages of human enrollees and pigs collected in this study (see SupplementaryTable 1, separate pdf).

**Supplementary Table 2**. The best-fitted model of substitutions based on AIC criterion for all segments of RVA, RVB, RVC and RVH used for maximum-likelihood phylogenetic tree constructions.

| **Gene** | **RVA** | **RVB** | **RVC** | **RVH** |
| --- | --- | --- | --- | --- |
| **VP1** | GTR+G4 | GTR+I+G4 | GTR+I+G4 | TIM+I |
| **VP2** | GTR+G4 | TIM2+I+G4 | GTR+I+G4 | GTR+I |
| **VP3** | GTR+I+G4 | GTR+I+G4 | GTR+G4 | GTR+I |
| **VP4** | GTR+I+G4 | GTR+I+G4 | GTR+I+G4 | GTR+I+G4 |
| **VP6** | GTR+I+G4 | GTR+I+G4 | GTR+I+G4 | GTR+I+G4 |
| **VP7** | GTR+I+G4 | GTR+I+G4 | GTR+I+G4 | TIM+I+G4 |
| **NSP1** | GTR+I+G4 | GTR+I+G4 | TIM3+I+G4 | TPM2+I |
| **NSP2** | GTR+I+G4 | GTR+I+G4 | TPM3u+G4 | TN+I |
| **NSP3** | GTR+I+G4 | GTR+I+G4 | GTR+I+G4 | TN+I |
| **NSP4** | GTR+G4 | TIM3+G4 | GTR+G4 | GTR+I+G4 |
| **NSP5** | HKY+I+G4 | GTR+I+G4 | TVM+G4 | TIM2+I |

**Supplementary Data Figure Legends**

**Supplementary Figure S1**.The geographical area that diarrhoeal patients and pigs were sampled over the study period. The map shows the Mekong Delta river region in southern Vietnam (including 6 provinces An Giang, Can Tho, Vinh Long, Dong Thap, Long An and Tien Giang), and Dong Thap province is the major study site. Red star indicates the Dong Thap Provincial Hospital where diarrhoeal cases in this study were attending. Purple circle represents the residential address of a human case enrolled from the study. The orange triangle represents the pig farms where porcine faecal samples were collected. The colour intensity correlates with the number of samples collected at that residence. The dotted green line highlights the Mekong Delta river that runs through the region and the green shaded area bounded within the line indicates the flooding area during the rainy season. The map scale bar is shown in the units of geometric km.

**Supplementary Figure S2**. The genotype constellation of each of the RVA strains identified in this study, using the online RotaC tool according to the guidelines of Rotavirus Classification Working Group.

**Supplementary Figure S3**. Maximum-likelihood phylogenetic trees inferred from the assembled nucleotide sequences for RVA VP1-VP3 and VP6 (A), and for NSP1-NSP5 segments (B). Trees are mid-point rooted and only bootstrap values of ≥75% are shown. Scale bars are in the unit of nucleotide substitutions per site. Strains were coloured according to the host species that the sequences were identified from.

**Supplementary Figure S4.** Maximum-likelihood phylogenetic trees inferred from the assembled nucleotide sequences for RVB VP1-VP4 and VP7 (panel A) and NSP1-NSP5 (panel B) segments. Trees are mid-point rooted and only bootstrap values of ≥75% are shown. Scale bars are in the unit of nucleotide substitutions per site. Strains were coloured according to the host species that the sequences were identified from.

**Supplementary Figure S5.** Maximum-likelihood phylogenetic trees inferred from the assembled nucleotide sequences for RVC VP1-VP4 and VP7 (panel A) and NSP1-NSP5 (panel B) segments. Trees are mid-point rooted and only bootstrap values of ≥75% are shown. Scale bars are in the unit of nucleotide substitutions per site. Strains were coloured according to the host species that the sequences were identified from.

**Supplementary Figure S6.** Maximum-likelihood phylogenetic trees inferred from the assembled nucleotide sequences for RVH VP1-VP4 and VP7 and NSP1-NSP5 segments. Trees are mid-point rooted and only bootstrap values of ≥75% are shown. Scale bars are in the unit of nucleotide substitutions per site. Strains were coloured according to the host species that the sequences were identified from.
