# Supplementary figures and images for: Unbiased whole-genome deep sequencing of human and porcine stool samples reveals circulation of multiple groups of rotaviruses and a putative zoonotic infection
Source: Virus Evol. 2016 Oct 3;2(2):vew027. doi: 10.1093/ve/vew027 (PMC5522372; doi:10.1093/ve/vew027)

Suppl Figure 1

The sampling area for diarrhoeal patients and pig farms in this study

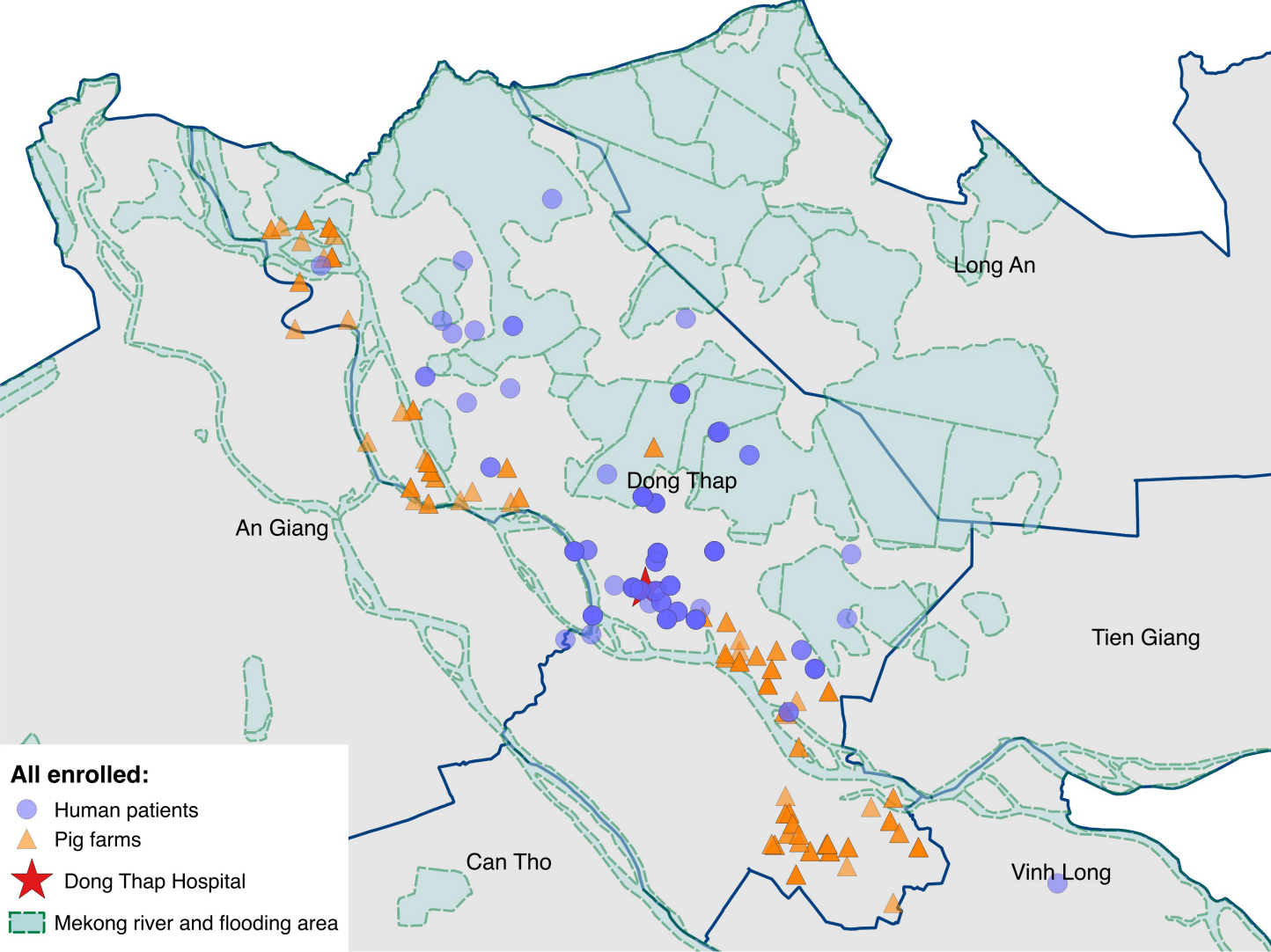

Supplement: Supplementary Data [file vew027_Supp.zip › SuppS1.pdf]

Suppl Figure 4A

Host

- Cow
- Human\_Other countries
- Pig\_Other countries
- Rat
- Sheep
- VIZIONS\_Pig

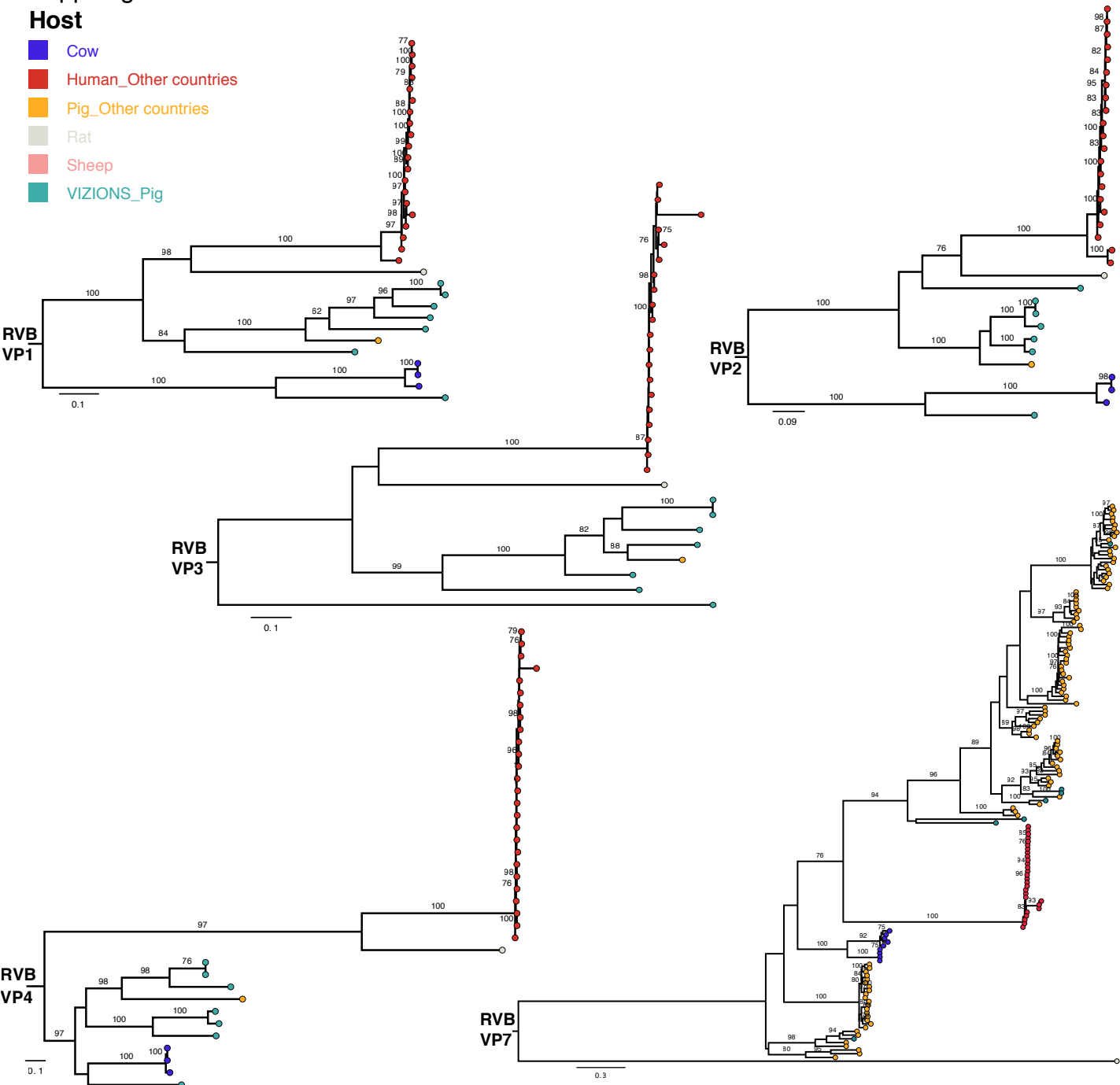

Supplement: Supplementary Data [file vew027_Supp.zip › SuppS4A.pdf]

Suppl Figure 4B

Host

- Cow
- Human\_Other countries
- Pig\_Other countries
- Rat
- Sheep
- VIZIONS\_Pig

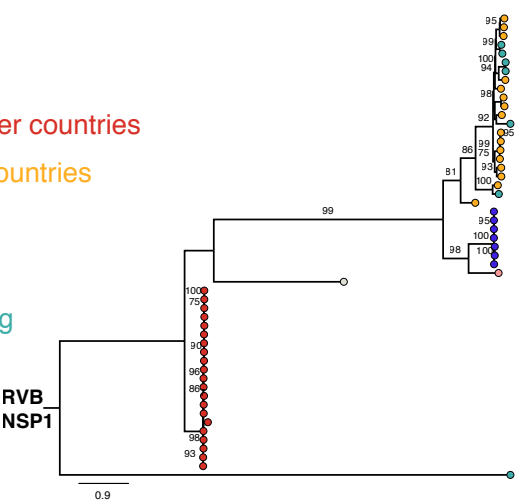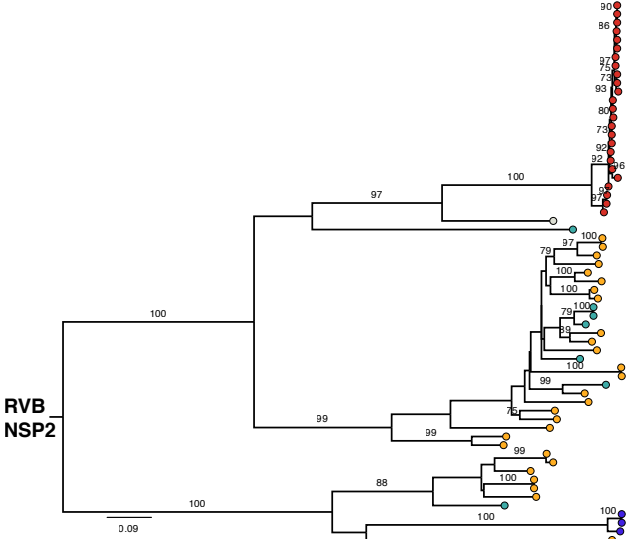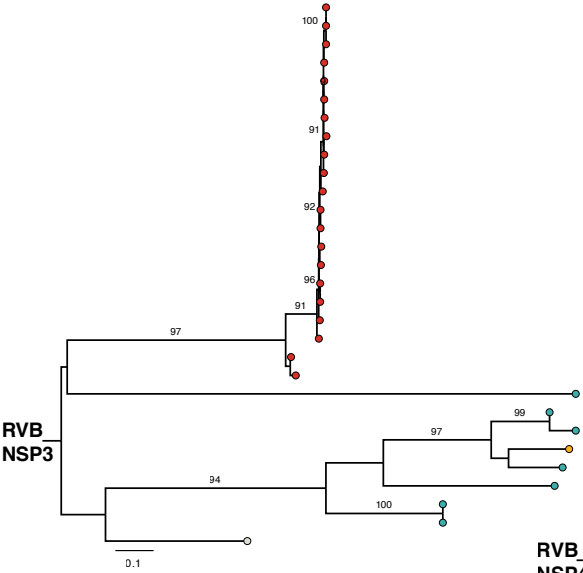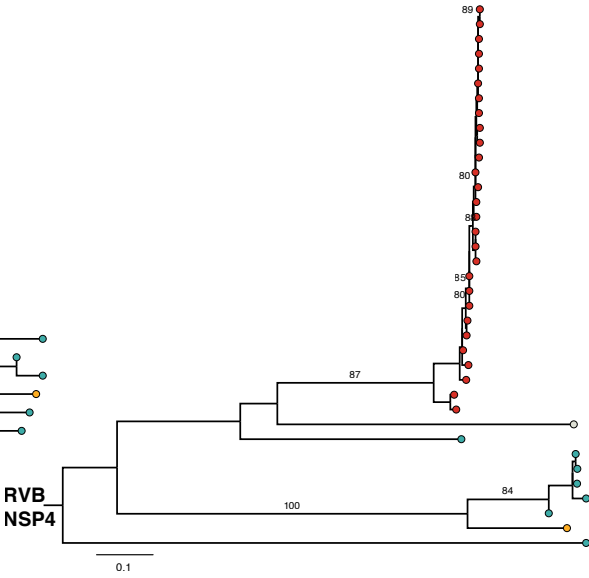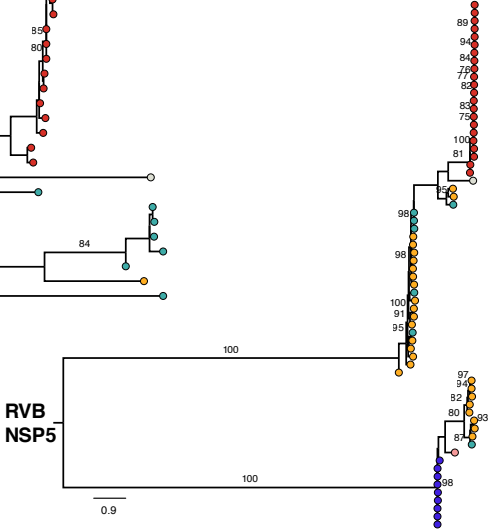

Supplement: Supplementary Data [file vew027_Supp.zip › SuppS4B.pdf]

Suppl Figure 5A

Host

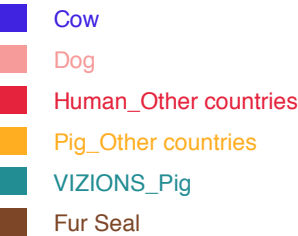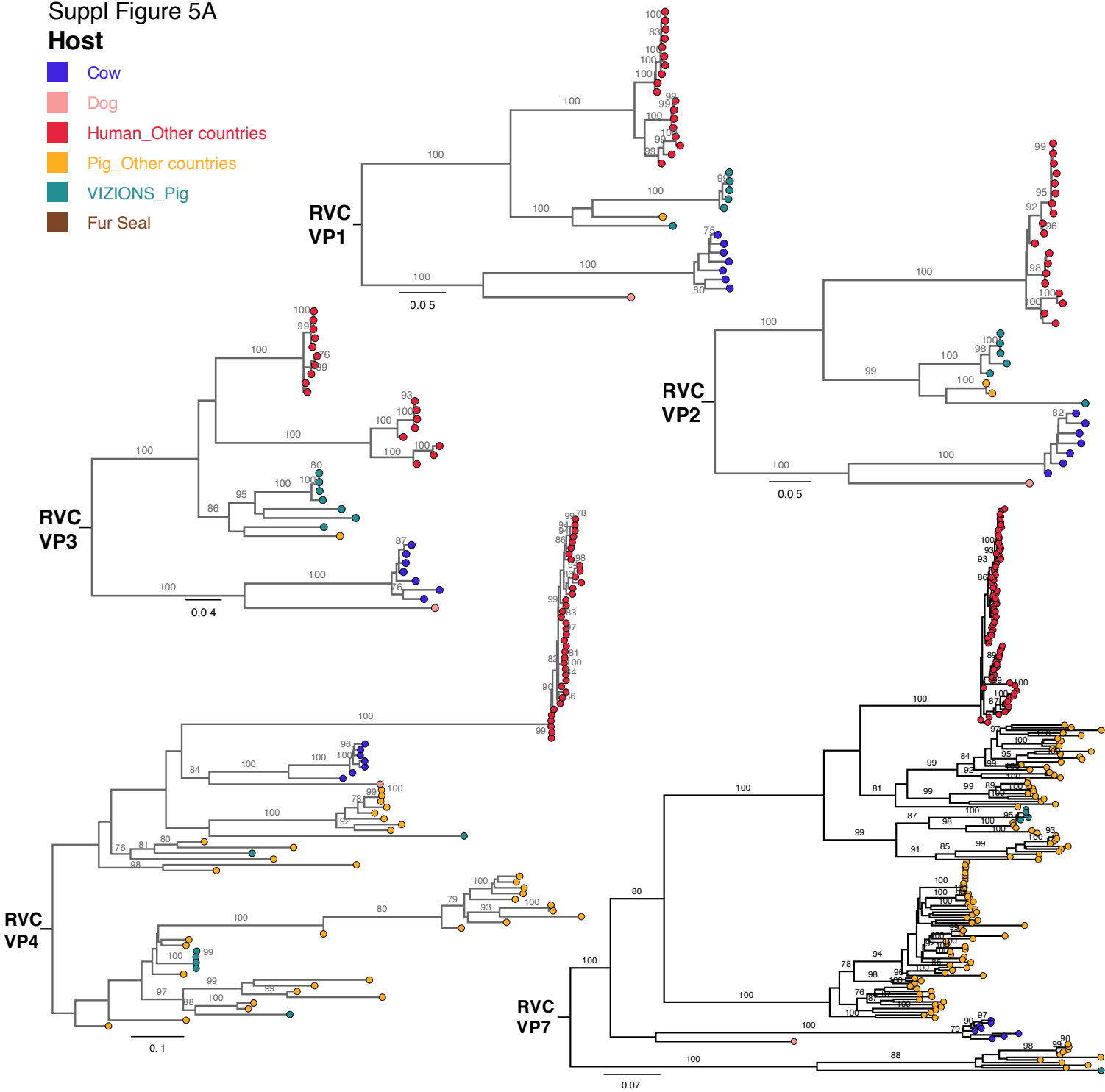

Supplement: Supplementary Data [file vew027_Supp.zip › SuppS5A.pdf]

## Host

 Dog

 Pig\_Other countries

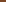 Fur Seal

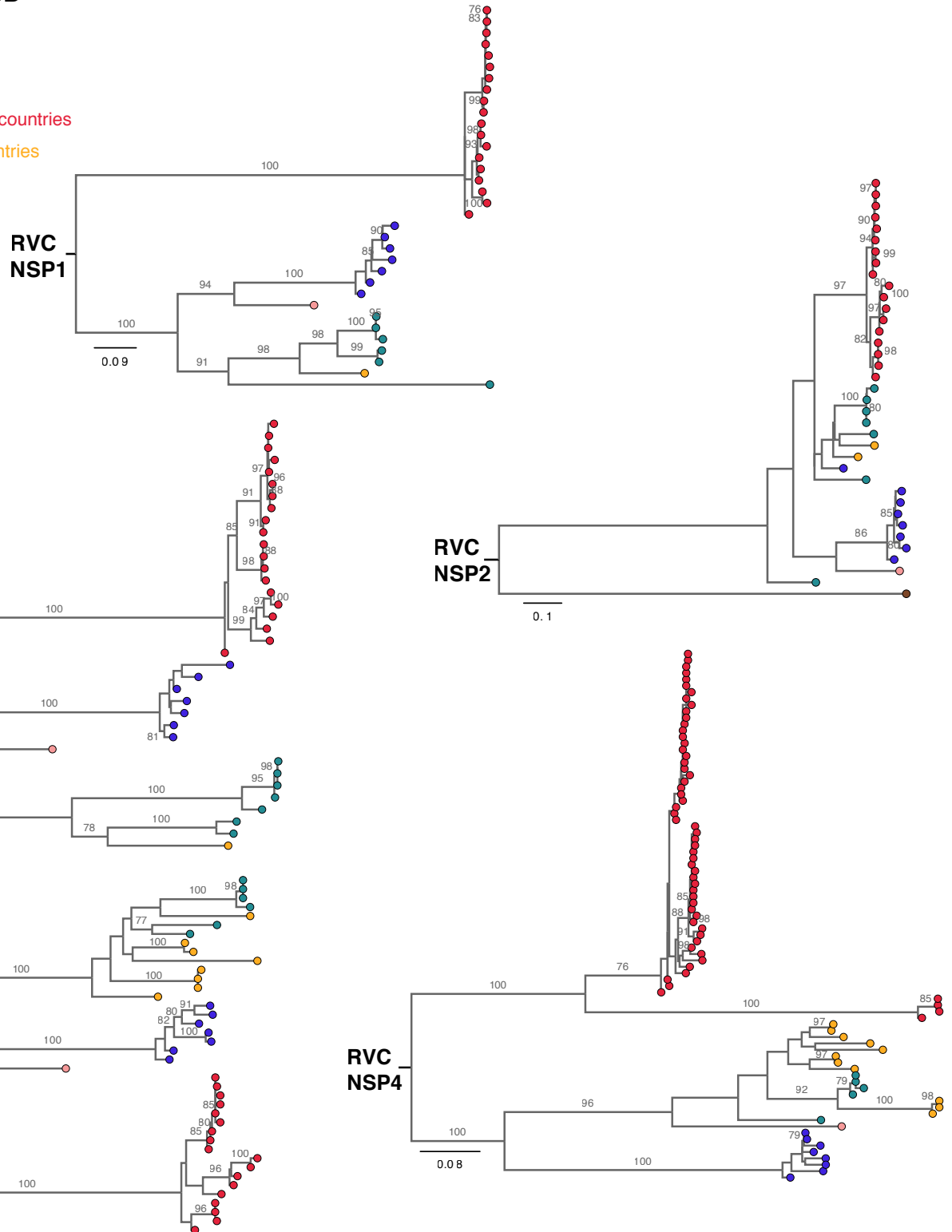

Supplement: Supplementary Data [file vew027_Supp.zip › SuppS5B.pdf]

# Suppl Figure 6

## Host

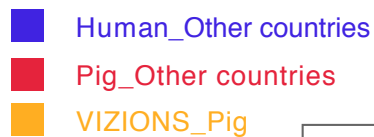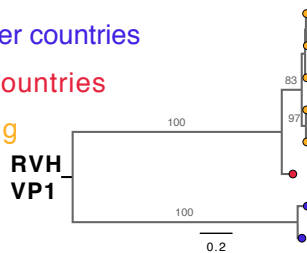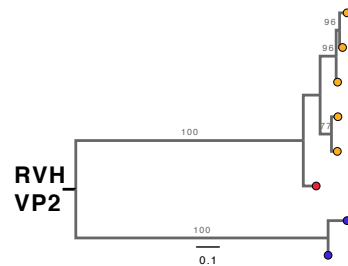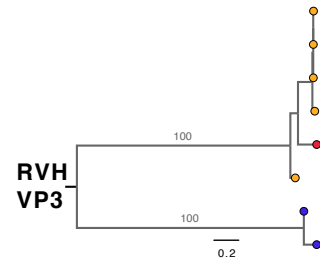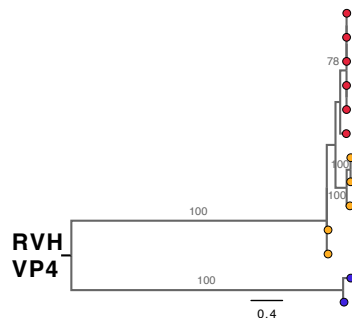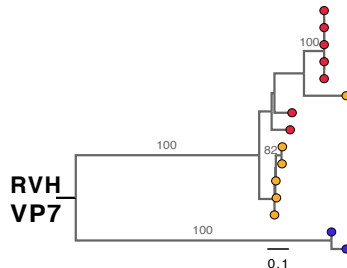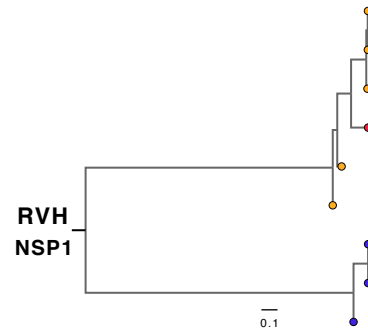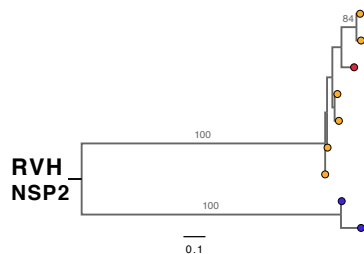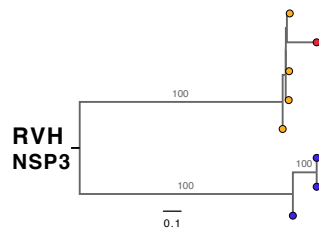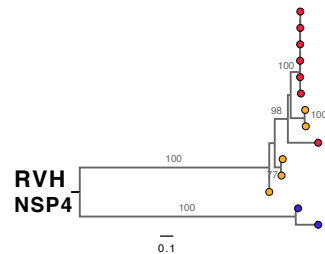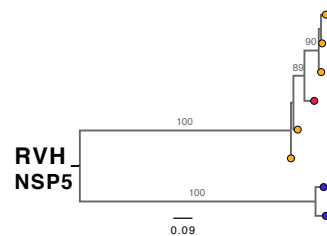

Supplement: Supplementary Data [file vew027_Supp.zip › SuppS6.pdf]
